# A Whole Food Plant-Based Approach for Migraine; A Case Series

**DOI:** 10.1177/15598276221120520

**Published:** 2022-08-20

**Authors:** Olga Morton, Mishkat Shehata, Nicole Gabbitas, Shireen Kassam

**Affiliations:** Calderglen Medical Practice, Hunter Health Centre, East Kilbride, Lanarkshire, UK (OM); Judges Close Surgery, West Sussex, UK (MS); Grimsby, UK (NG); and King’s College Hospital and King’s College London, 8629 University of Winchester, Hampshire, UK (SK)

**Keywords:** diet, nutrition, lifestyle, plant-based, migraine

## Abstract

Migraine is a common headache disorder that adversely affects quality of life. It is usually considered a chronic lifelong disorder. Although several treatments are available, their benefits need to be considered with knowledge of potential side-effects and it is rare for people with migraine to be free of attacks. There are some well known dietary and lifestyle triggers of migraine and patients are usually keen to explore lifestyle approaches to manage the condition. Given the lack of high quality studies, physicians tend not to emphasize these lifestyle approaches. Therefore, many patients are left to explore these options for themselves. There is a growing body of evidence to support the role of a healthy diet, along with elimination of alcohol and reduction in coffee intake, in the prevention and treatment of migraine. Here, we report 3 cases of the successful treatment of migraine using a whole food plant-based diet, resulting in significant reduction or elimination of medication use.

‘Coffee and alcohol are well known triggers for migraines and our three cases reduced or eliminated these to varying degrees’.

## Introduction

Migraines affect 1 in 10 people worldwide and the incidence is on the rise.^1^ Migraines are characterized by attacks of moderate or severe unilateral headache and associated symptoms such as photophobia, phonophobia, nausea and vomiting and/or pre-migraine aura. Although not a fatal disease, migraine ranks as the sixth highest cause of disability.^2^ In women, migraine attacks tend to be longer, with a pattern of increased risk of headache recurrence and greater disability.^3^

Successful migraine treatment is defined as a decrease in the duration of the attacks or a 50% reduction in the number of migraine attacks per month, following acute therapy.^4^

Medications are helpful but the efficacy is often limited, associated with side-effects and undesired consequences, such as headaches due to medication overuse. More evidence has been emerging that lifestyle and dietary interventions may offer a very promising approach in the treatment of migraine.

A number of foods have been identified as triggers of migraines such as chocolate, coffee, cheese, alcohol or citrus fruit.^5^ Various dietary approaches have been reported in the literature including low-fat,^6^ vegan^5^ and ketogenic diets.^7^ However, there remains limited high quality data to inform clinical practice and in general most patients don’t receive dietary counselling.

Plant-based diets are gaining popularity given the well-described benefits for improving cardiometabolic health, established safety and the co-benefits for planetary health. A recent case report described the dramatic and rapid remission of migraine with a nutrient-dense whole food plant-based diet (WFPBD).^8^

We present 3 case reports where duration, frequency and intensity of migraines significantly improved in all 3 patients after transitioning to a WFPBD, gradually eliminating all processed foods and most animal products (dairy, red meat, poultry, eggs) but also milk chocolate, oil and sugar. Various fruits and leafy vegetables, legumes, wholemeal pasta, brown rice, pulses, nuts and seeds were encouraged on a daily basis. For each case, the dietary pattern before and after adopting a WFPBD is summarized in Table 1 and a summary of duration, severity and intensity of migraines can be found in Table 2.

**Table 1.** Dietary Pattern Before and After Adopting a Whole Food Plant-Based Diet.

Usual diet before WFPBD	Case 1	Case 2	Case 3
Breakfast	Biscuits, porridge or toast	Cereal with cow’s milk or toast and banana	Coffee or tea with lactose-free cows’ milk and sugar
Lunch	Soup or sandwich for lunch with sweets or cake and occasional crisps	Sandwiches with cheese or cold meat with fruit or salad	Cheese sandwich with crisps and fruit juice
Dinner	A processed ready meal for dinner or a home-cooked meal with meat as the bulk of the meal	Pasta, rice, potatoes, with vegetables and meat or fish for at least 5 days/week	Seafood, fish or chicken with vegetables and white rice or bread
Snacks	Crisps, fruit, cakes	Fruit, nuts, crisps, cake	Pastries, sweets, crisps and chocolate
Adopted WFPBD			
Breakfast	Fruit salad (melon, kiwi, grapes and pomegranate)	Overnight oats with seeds, grated carrot, apple, grapes, banana, turmeric and oat-milk	Green ginger and mint smoothie or chia oats with oat-milk and berries
Lunch	Greek yoghurt, blueberries and honey for lunch	Rice or pasta dish with sauce and vegetables, lentils, beans and tofu	Tomato, avocado and chickpea bruschetta on rye sourdough bread
Dinner	Veggie chilli, baked sweet potato with occasional fish	a rice or pasta dish with sauce and vegetables	Crispy tofu ramen with vegetables and noodles
Snacks	Mixed fruit and nut and dark chocolate	Dates, nuts, fruit and vegan 70% chocolate	Fruits and vegetables, nuts and seeds

**Table 2.** Duration, severity and intensity of migraines.

	Before WFPBD			After WFPBD			Length of follow-up
	Duration	Frequency	Intensity	Duration	Frequency	Intensity	Months
Case 1	8 hours	30/month	10/10	8 hours	10/month	3–4/10	10
Case 2	5 days	3–4/month	8/10	1 day	1/month	3–4/10	48
Case 3	3 days	3–4/month	8/10	None	None	0/10	24

## Case 1

A 47-year-old, White, European female presented following a self-referral to a lifestyle medicine doctor with a 6-year history of migraines with aura. The migraines occurred daily with a prodrome of confusion, brain fog, visual floaters, tinnitus and vomiting. The pain was experienced in the right shoulder radiating into the right posterior neck, and always ending as a dull ache behind her left eye. During one severe episode, the patient was admitted to hospital where imaging studies were unremarkable. Migraines tended to be worse around menstrual periods but oral contraception was not beneficial. Over a six-year period, the patient was unable to work as a lawyer for up to 5 months per year, often cancelling social arrangements and unable to drive due to vision disturbances.

Initial treatment was pharmacological with acute phase medication (aspirin, indomethacin and triptans). Preventative medications advised by her neurologist (propranolol, sodium valproate, pregabalin, topiramate, amitriptyline and candesartan) had been tried for 2 years and were ineffective. Occipital nerve block initially reduced the frequency of migraines; however, it soon became ineffective and further occipital nerve blocks were not successful. Botox injections were initially successful in reducing the frequency of migraines to 14 out of every 30 days, but were subsequently ineffective. A regular use of a neuromodulating device helped in reducing severity and frequency, and migraines were well-managed for 1 year. Unfortunately, it became unavailable due to manufacturing problems during the COVID-19 pandemic, leading to daily frequency of migraines for 3 continuous months, until finally a WFPBD was introduced over a 4 week period.

Coffee intake was gradually reduced from 5–6 cups to zero cups per day, and replaced with herbal tea. Alcohol was not consumed before or after changing to WFPBD. Water intake increased from 1 to 1.5 L/day. In addition, attention was paid to sleep hygiene, aiming for 8 hours per night and a minimum of 30 minutes of walking per day was subsequently incorporated into her daily routine. Four weeks after the introduction of a WFPBD, the frequency of headaches reduced, with no medication or any other conventional treatments required subsequently.

The patient was able to return to work initially part-time, and full-time 1 month later. The patient also reported more energy, clearer skin and improvement in mental health. There was a marked weight loss of 8 kg in 2 months, reducing her body mass index (BMI) from 29 to 26.

When 4 months later the patient had a rebound after returning to her previous diet, her headache frequency increased from 2.5 to 4–5 per week. After returning to a WFPBD, she was able to achieve the same benefit again. She has been following a WFPBD for the last 10 months with no need for preventative medication and only occasional use of frovatriptan (once every 2 months).

## Case 2

A 49-year-old, White, European female had an 18-year history of migraines. Initially the headaches followed her menstrual cycle but later on, the headache frequency increased to 3–4 times per month. They started with visual aura and hypersensitivity to sounds, absent-mindedness, followed by a ‘vice-like pressure’ above the left eyebrow and pain behind the left ear. After lifting of the pain and pressure, the patient experienced 2 days of reduced cognitive functioning (not remembering names, commitments, routines) and a sense of emptiness. She was not able to enjoy her young family, experienced difficulty carrying out a busy profession as a secondary school teacher and often had to cancel her social engagements.

After approaching her family doctor, she was told to take ibuprofen on the days of headaches, which did not reduce the pain severity or frequency. Disappointed with the medical profession, she did not pursue further medical treatments.

Iron deficiency anaemia was diagnosed at the age of 23 and recurred during 3 pregnancies, requiring treatment with iron supplements. Gestational diabetes was diagnosed during her third pregnancy but this did not require medication. During the last 3 years prior to the transition to the WFBPD, she experienced typical symptoms of type 2 diabetes (constant thirst, polyuria, lethargy). She did not consult a doctor, but diabetes awareness and the fear of living with possible complications triggered the change in her diet. The diet change was self-initiated after an extensive period of online research without direct consultation with a health professional.

Twelve weeks after transitioning to a WFPBD, the severity and the frequency of migraines had decreased, and the usual aura did not develop into a migraine anymore. If it did, once every 3 months, it was usually associated with eating dairy, sugar or processed foods and it was much less severe, lasting for a maximum of 3 days. She did not drink coffee or alcohol before or after changing her diet and water intake remained the same at 1.5–2L daily. In addition, attention was paid to sleep hygiene, aiming for 8 hours per night and a minimum of 30 minutes of walking per day was subsequently incorporated into her daily routine.

She has been following a WFPBD for the last 4 years with only occasional relapses. She is now able to enjoy her family life, carry on with teaching and to live without analgesia. Her sleep, energy levels and mental health have improved. She has reduced her BMI from 29 to 22, experiencing healthier looking skin and overall enjoys a better quality life.

## Case 3

A 33-year-old doctor of Arab ethnicity presented with a 16-year history of migraines. These started at the age of 17 with a frequency of up to 4 times per month. The pain was usually behind the right eye, associated with nausea, vomiting, phonophobia, photophobia, flashing lights, followed by a temporary lack of vision in the right eye. The migraines impaired her ability to concentrate, affecting university studies and her work schedule later in life.

Paracetamol and sumatriptan were used for the acute attacks, and propranolol and amitriptyline were used for prevention. She was also diagnosed with polycystic ovarian syndrome and irritable bowel syndrome (IBS) aged 23 and long-COVID aged 33.

Given her profession as a doctor, she undertook a period of extensive research from online and medical organizational resources and made the decision to transition to a plant-based diet. Four weeks into transitioning to a WFPBD, the migraines had discontinued. Coffee was not excluded but its consumption was limited to before 3pm, alcohol consumption was eliminated and her water intake was increased from 2 glasses to 2 L/day. Meditation, high intensity interval training exercise, weight training and yoga were also practised on a regular basis. She also received cognitive behavioural therapy for stress-triggered migraines. A significant improvement was noted in life quality, positive mindset, sleep and stable energy levels even with a diagnosis of long-COVID. She lost weight and reduced her BMI from 29 to 22. Her constipation and IBS symptoms also resolved. She has been following an exclusively WFPBD for 2 years with no recurrence of migraine.

## Discussion

This small case series reports the potential role of a WFPBD for treatment of migraines, with concurrent, significant weight loss. The 3 females described all had a long history of migraines, which were poorly controlled with conventional pharmaceutical approaches and associated with impaired quality of life. It is worth noting the 2 different ethnicities represented by the 3 cases. There may be differences in migraine prevalence and treatment response amongst different ethnicities but certainly, within the UK, there has been little research in this area.^9^

Previous studies have suggested a benefit for migraine of a healthy plant-based diet centred around fruit, vegetables, whole grains and beans. A 16-week randomized study of 42 people with migraine reported a reduction in headache pain, frequency and intensity with a low-fat vegan (plant-based) diet.^5^ In addition, a recent case report described the impressive reversal of migraine with a WFPBD emphasizing the consumption of dark green leafy vegetables.^8^ In a cross-sectional study of 266 women with migraine, adherence to a healthy plant-based diet was associated with up to a 60% reduction in severity, disability and duration of headaches. In contrast, an unhealthy plant-based diet centred around refined grains, sugar and processed foods was associated with greater severity and disability.^10^

It is thought that inflammation and oxidative stress are key mechanisms involved in the development of migraines.^11^ A WFPBD emphasizes foods that are high in anti-inflammatory and anti-oxidant compounds^12^ and eliminates foods that are pro-inflammatory or trigger migraines, such as cheese and food preservatives and additives.^13^ A WFPBD has a well-documented ability to reduce inflammation and oxidative stress, thus providing plausible mechanisms for the observed clinical improvements.^14^ In addition, a WFPBD is very effective at supporting the maintenance of a healthy body weight and weight loss may have also contributed to the improvement in symptoms in the 3 cases described.^15^ Weight loss can be achieved by various dietary approaches, but the advantage of a WFPBD is the associated benefits for long-term cardiometabolic health which is not seen with other dietary approaches such as a low-carbohydrate or ketogenic diet.^16^

Certain plant-based foods have also been reported to triggered migraines, with a recent study highlighting a negative impact of consuming watermelon.^17^ In our three cases, there were no plant-based foods identified as triggers for migraine. Coffee and alcohol are well known triggers for migraines and our three cases reduced or eliminated these to varying degrees. Two of the three individuals increased hydration through increased water consumption, which is known to be beneficial for migraine.^18,19^

There is some evidence to suggest that reducing the consumption of omega-6 fatty acids and increasing the ratio of omega-3 to omega-6 fatty acids may be of benefit for people with migraine by altering blood levels of biochemical mediators of pain.^20^ Our cases naturally reduced consumption of omega-6 fatty acids through minimizing the consumption of processed foods and it may be worth emphasizing plant sources of omega-3 fatty acids such as flaxseeds and chia seeds and also considering an algal omega-3 fatty acid supplement when transitioning to a plant-based diet for migraine management. Other supplements that have been shown to benefit people with migraine include magnesium,^21^ riboflavin^22^ and coenzgyme Q10^23^ by reducing inflammation and oxidative stress.

Addressing all pillars of lifestyle medicine is important for managing migraine given that lack of sleep and stress are known triggers.^24^ In addition, although many people with migraine may actively avoid physical activity,^25^ it is thought to be beneficial for migraine prevention.^26^ Case 3 embraced several aspects of lifestyle medicine for migraine management, whereas cases 1 and 2 used a dietary approach first and, once improvement in migraine was achieved, incorporated further healthy habits. A WFPBD alongside a lifestyle medicine approach is also attractive given the fact that people with migraine have an increased risk of comorbidities including cardiovascular disease, inflammatory conditions, depression and anxiety.^27^ Even though further high quality studies would be beneficial,^28^ including in male migraine sufferers and people of different ethnicities, there are no side-effects with this approach and our report highlights the significant reduction in frequency and intensity of headache, improvement in quality of life and remarkable weight loss that is possible for people with chronic migraine.
